# Construction and Application of an Information Closed-Loop Management System for Maternal and Neonatal Access and Exit Rooms: Non Randomized Controlled Trial

**DOI:** 10.2196/66451

**Published:** 2025-04-07

**Authors:** Shafeng Jia, Naifeng Zhu, Jia Liu, Niankai Cheng, Ling Jiang, Jing Yang

**Affiliations:** 1 Department of Obstetrics, The Affiliated Suzhou Hospital of Nanjing Medical University, No. 26 Daoqian Street, Gusu District, Suzhou, Jiangsu, 215008, China, 86 18626285408; 2 Department of Obstetrics and Neonatology, The Affiliated Suzhou Hospital of Nanjing Medical University, Suzhou, Jiangsu, China; 3 Department of Nursing, The Affiliated Suzhou Hospital of Nanjing Medical University, Suzhou, Jiangsu, China

**Keywords:** mother-infant same-room management, information-based identity verification, closed-loop management system, newborn safety management

## Abstract

**Background:**

Traditional management methods can no longer meet the demand for efficient and accurate neonatal care. There is a need for an information-based and intelligent management system.

**Objective:**

This study aimed to construct an information closed-loop management system to improve the accuracy of identification in mother-infant rooming-in care units and enhance the efficiency of infant admission and discharge management.

**Methods:**

Mothers who delivered between January 2023 and June 2023 were assigned to the control group (n=200), while those who delivered between July 2023 and May 2024 were assigned to the research group (n=200). The control group adopted traditional management methods, whereas the research group implemented closed-loop management. Barcode technology, a wireless network, mobile terminals, and other information technology equipments were used to complete the closed loop of newborn exit and entry management. Data on the satisfaction of mothers and their families, the monthly average qualification rate of infant identity verification, and the qualification rate of infant consultation time were collected and statistically analyzed before and after the closed-loop process was implemented.

**Results:**

After the closed-loop process was implemented, the monthly average qualification rate of infant identity verification increased to 99.45 (SD 1.34), significantly higher than the control group before implementation 83.58 (SD 1.92) (*P*=.02). The satisfaction of mothers and their families was 96.45 (SD 3.32), higher than that of the control group before the closed-loop process was implemented 92.82 (SD 4.73) (*P*=.01). Additionally, the separation time between infants and mothers was restricted to under 1 hour.

**Conclusion:**

The construction and application of the information closed-loop management system significantly improved the accuracy and efficiency of maternal and infant identity verification, enhancing the safety of newborns.

## Introduction

In the modern medical environment, ensuring the safe movement of newborns and mothers and effectively managing room exits and entry is an important part of obstetric care and newborn health management. In the 2022 Safety Goals for Patients and the Action Plan to Improve Maternal and Infant Safety, the China Hospital Association clearly emphasized the importance of strengthening maternal and newborn safety. Strategies include improving neonatal access management systems, implementing continuous quality improvement measures, and enhancing safety protocols [1-3]. However, studies have shown that patient identification error rates in medical settings remain as high as 20% despite strict reconciliation procedures [4]. Intelligent systems play an important role in the treatment and care of diseases [5,6]. As the scale of the hospital and the number of beds increases, the traditional manual records and management methods can no longer meet the demands for efficient and accurate management; an information-based and intelligent management system is urgently needed to optimize this process [7]. Although information technology has been widely used in patient identity verification, there are limited reports on its application in identity verification and door access time monitoring in the neonatal ward. The infant room entry and exit information management system for mother-infant co-care uses modern information technology and ‘Internet of things’ devices to achieve full process monitoring and management of an infant’s movement from the rooms [8]. It uses radio-frequency identification tags, two-dimensional code scanning, and biometric identification technologies to automatically record and track an infant’s entry and exit information and achieve closed-loop management. Implementing a full-process closed-loop management system is a core strategy of modern nursing quality management that can improve the intelligent neonatal care. Starting from July 2023, our hospital implemented identity verification and door access–time monitoring for the infant entry and exit rooms based on the mobile nursing platform and implemented closed-loop information management. This study expanded the application of information technology in the field of newborn identity verification and explored the effectiveness of closed-loop management in newborn care, which was an important supplement to the field of intelligent neonatal care. This study aimed to construct an information closed-loop management system to improve the accuracy of identification in mother-infant rooming-in care units and enhance the efficiency of infant admission and discharge management.

## Methods

### Study Setting

In July 2023, the information closed-loop management system for barrier-free neonatal wards was officially launched in the Obstetrics and Gynecology ward of the hospital. The study included 400 mothers (n=200 per group) admitted to the hospital between January 2023 and May 2024. The exclusion criteria were as follows: the mothers needed to be transferred to another hospital after admission, the medical records were incomplete, or the newborns needed to be isolated from the mother due to illness. The parturients admitted to the hospital between January 2023 and June 2023 were assigned to the control group, while those admitted between July 2023 and May 2024 were assigned to the research group. Data on metrics such as the workload and work efficiency of nurses, the satisfaction of mothers and their families, the standard rate of newborn identity verification, and newborn visit time qualification rate were collected and statistically analyzed.

### Information Closed-Loop Management System for Mother and Infant Newborns

Manual inspection methods are used in the traditional newborn admission and discharge management process. These methods rely on nursing staff to visually compare the wristband and foot information of the mother and the newborn and to conduct oral checks on the following main content: (1) room information verification, (2) oral confirmation, and (3) postadmission information verification. This method mainly relies on nursing staff or inquiries from the mother and her family, where the human factor has a large impact. Reports show that 40‐60% of mothers experience postpartum fatigue, which manifests as decreased attention during medical examinations and communication, thereby increasing the risk of verification errors and medical accidents [9-11].

### Design of a Closed-Loop Management System for the Neonatal Access Room

The closed-loop management system includes three functional modules: (1) the medical order module, which includes the medical order system and the electronic nursing system; (2) the label barcode printing module, which prints maternal wristbands and newborn ‘A and B’ wristbands; and (3) the statistical function module, which manages the statistical room time pass rates.

The management system includes a database management system (DBMS), handheld personal digital assistant (PDA) devices, and QR barcode recognition technology. PDAs equipped with a barcode scanner can print and scan the QR codes, which serve as identifiers, to read and transmit data to the central database server through a wireless network. The DBMS is responsible for processing data requests from the PDA device. During the management of newborn entry and exit, the PDA device is used to verify whether the QR code matches the DBMS records. If the match is successful, the server sends the confirmation information back to the PDA device and displays the correct identification details along with a voice prompt on the PDA device, indicating successful authentication. Simultaneously, the interface records the job number of the nursing staff and the time of entering and leaving the room for process management. It is worth noting that only authorized caregivers can use the PDA device for identification, ensuring the security of the system and avoiding mistrust. This management system provides a fast, accurate, and safe method for confirming the identity of the mother and child, thereby improving the efficiency of hospital work and ensuring the safety of the newborn.

### Management Plan for Building a Closed-Loop Information System for Newborn Admission and Discharge

**Figure 1.**
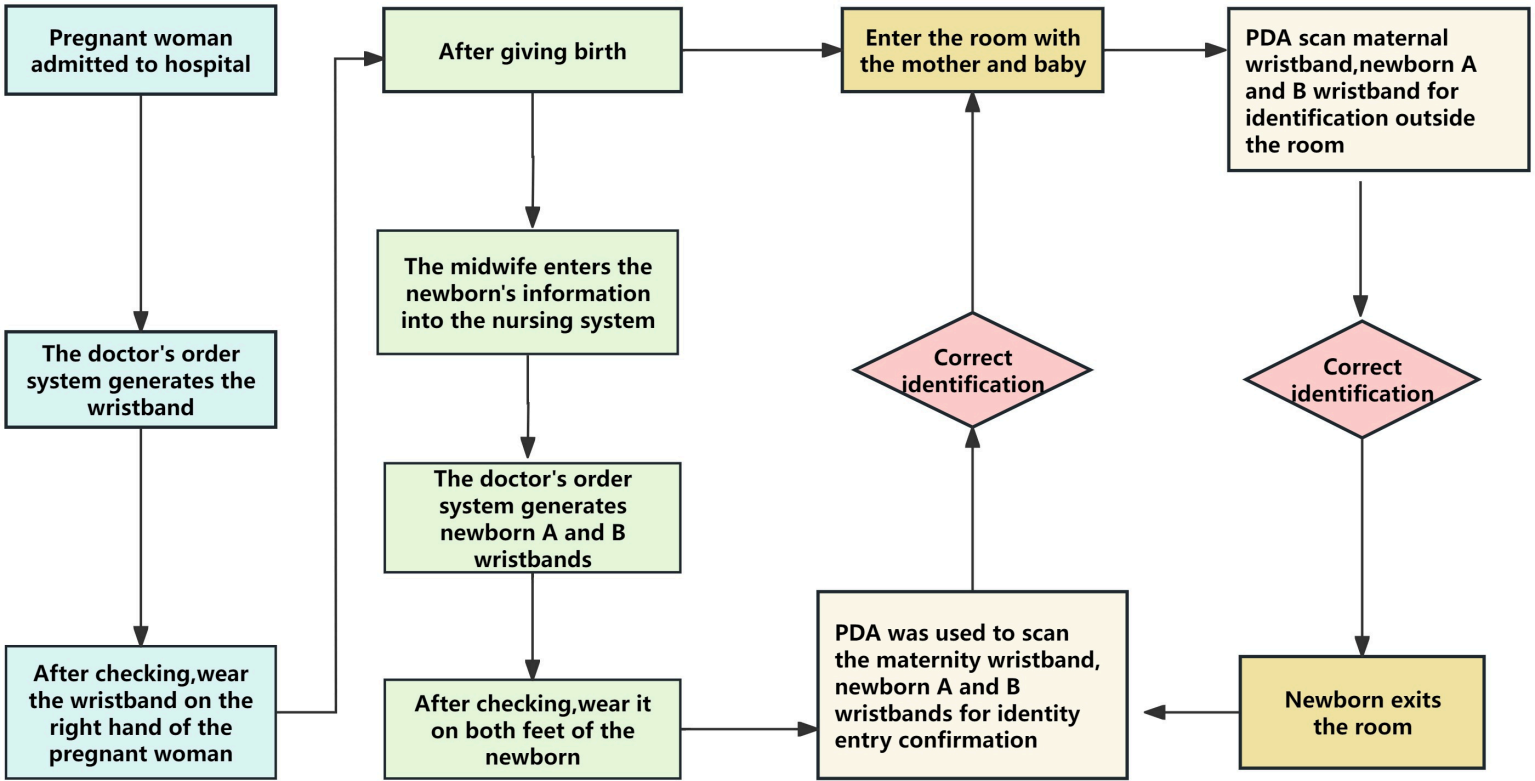
Identification process for neonatal admission and discharge. PDA: personal digital assistant.

The management team comprised five members, including one leader, with the deputy director of the nursing department responsible for leading the entire closed-loop management work. The team members organized the neonatal exit and entry operation through multiple surveys and consulted relevant literature [12-17]. In conjunction with the Information Department, they designed the closed-loop management system for neonatal exit and entry with the objective of monitoring and controlling the entire process, to ensure the safety of children. The process uses barcode technology, a wireless network, mobile terminals, and other information technology equipment to facilitate closed-loop management of newborn exit and entry. Furthermore, the team secured a computer software copyright (certificate number: 11934939). The specific operational process is shown in Figure 1. This flowchart describes the closed-loop information management process from the time the newborn is separated from the mother to the time of returned to the mother, covering admission, through delivery, and the mother’s discharge. The process is divided into the following steps:

1. Pregnant women complete admission registration in the outpatient system, generating accurate personal information based on their identity card or medical insurance card, including name, age, and unique hospitalization number.
2. Pregnant women enter the ward and use the ward system to generate wristbands, which are verified by two staff members before being worn on the right hand, include details such as name, hospitalization number, age and a QR code.
3. After the baby is delivered, the system imports delivery information and generates neonatal A and B wristbands with QR codes, which are verified by two staff members, and worn on the wrists and feet of the newborn.
4. When the mother and newborn leave the delivery room, a PDA device is used to scan the QR code on the wristbands of the mother and newborn for identification.
5. When the mother and infant enter the room, a ward nurse uses a PDA device to scan the QR code on the wristband of the mother and newborn again for identification to form a closed loop of admission and exit.
6. When the newborn leaves the mother for examination, vaccination, or treatment, a PDA device is used to scan the QR code on the mother and the newborn’s wristband for identification in the exit room.
7. When returning to the mother, a PDA device is used to scan the wristbands of the mother and newborn again to check the QR codes at the admission identification site to form a closed loop of access. The entire process emphasizes closed-loop management, ensuring the safety and correct identification of mothers and infants during hospitalization and improving the quality and efficiency of medical services.

### Identity Verification of Pregnant Women’s Admission Information

After the pregnant woman is admitted to the hospital, the identity verification process follows three steps: (1) completion of hospitalization registration: the system automatically assigns a unique hospitalization number as the pregnant woman’s identifier within the hospital; (2) wristband printing: after the pregnant woman is transferred to the ward, the nursing staff initiates the wristband printing function through the medical order system. The wristband is printed with the name, gender, time of admission, age, the hospital number of the pregnant woman, and a unique information QR code; (3) review: before wearing the wristband, the nursing staff strictly review the printed information and the actual details of the pregnant women to ensure the consistency of information.

### Newborn Identity Verification After Delivery

After delivery, the newborn identity verification process is as follows: (1) information entry: the midwife records the basic information of the newborn on the mobile nursing platform in the delivery room or operating room, including birth time, gender, and weight; (2) newborn wristband generation: the maternal personal medical consultation system generates the corresponding newborn A and B wristbands comprising the newborn’s information. The wristband is printed by a barcode printer with information including the name of the pregnant woman, sex of the newborn, weight, hospital number, and QR code; (3) information check: the midwife performs a double-check to ensure that the wristband details match those of the newborn and the mother; (4) wristband wearing: after examination, the midwife attaches A and B wristbands to the newborn’s ankles and informs the mother and family of their significance, emphasizing that they should not be removed to ensure the safety of the newborn; (5) identity matching: before the mother is transferred to the mother-baby room, the midwife scans the mother’s and the newborn’s A and B wristbands. If the PDA device shows a match, the identification is confirmed, and both are safely transferred to the room.

### Verification of Maternal Identity in the Mother-and-Baby

To ensure the safety of the mother and child within the same room, the following strict identity verification process is implemented: (1) handover and information check: the midwife and nursing staff conduct a detailed handover according to the protocol, verifying the basic information of the mother and newborn and record them; (2) PDA scan to verify identity: the mother and child use the PDA device in the same room to scan the mother’s wristband and the two-dimensional QR codes of the A and B newborn wristbands, ensuring identity consistency; (3) authentication completion: if the PDA indicates a successful match, the authentication process is considered complete.

### Identity Verification During Newborn Entry and Exit from the Mother-and-Baby Room

**Figure 2.**
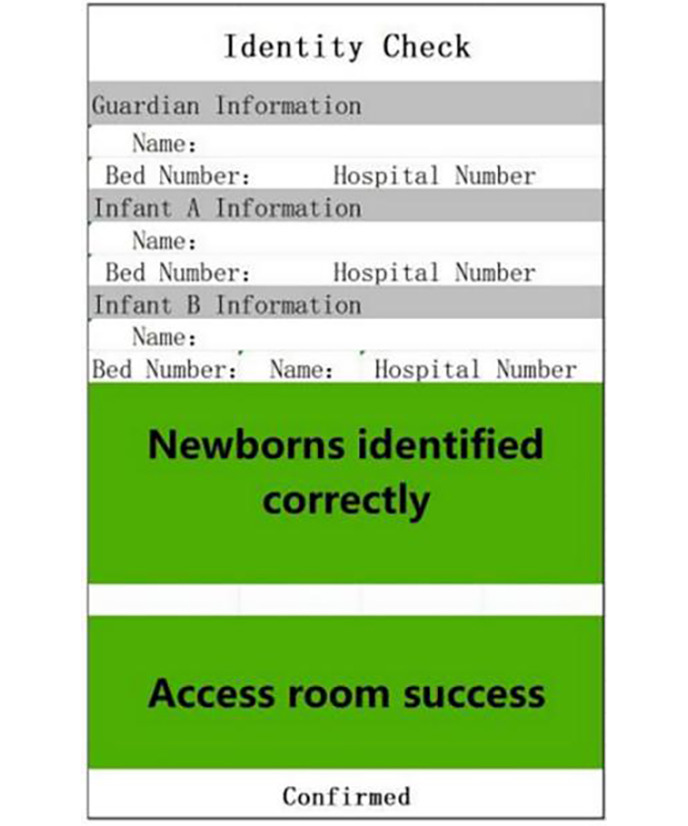
The system successfully identifies and confirms the mother-infant identity match.

Newborns often need to be temporarily separated from their mothers for routine nursing activities such as morning baths, examinations, and vaccinations. In this context, the safety risks are increased as multiple newborns may be under the custody of the nursing staff at the same time [18]. Accurate identity verification plays a vital role in ensuring the safety of newborns and avoiding identity confusion. The following identity verification process is executed: (1) identification before leaving the room: nursing staff use PDA equipment to scan the A and B wristbands of mothers and newborns to confirm the identity information. The PDA device confirms the identity-matching with the green module and voice prompt, and displays the job number of the executor and the time of the newborn’s exit (Figure 2); the nurse confirms the information with the mother and family members to ensure accuracy; (2) post-entry verification: when the newborn is returned to the ward after completing the relevant project activities, the nursing staff scan the QR code of the mother and newborn again. Similarly, the PDA device verifies the identity and records the job number and the entry time of the executor to complete the entry verification.

**Figure 3.**
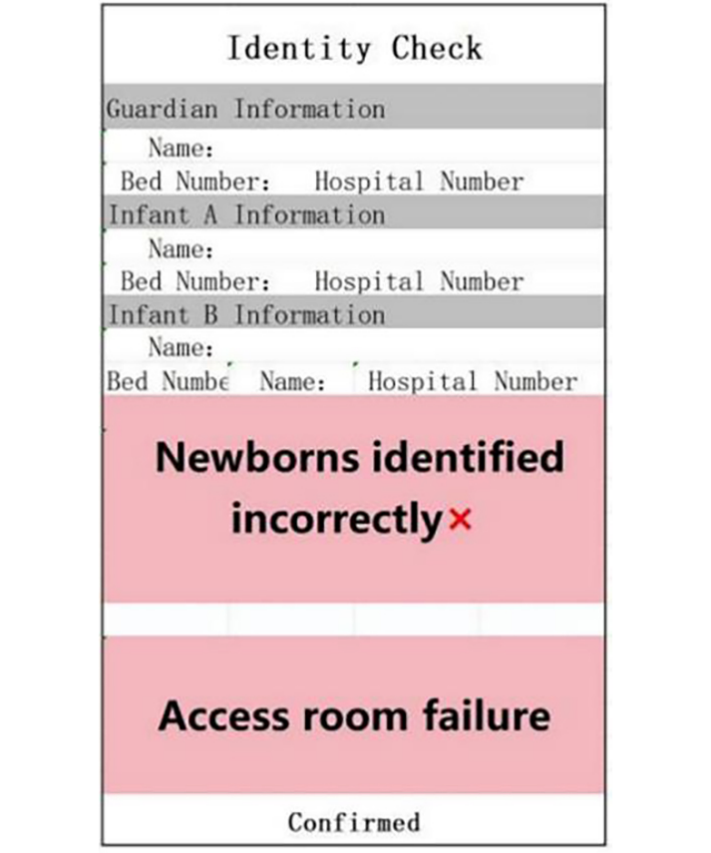
Identification failures due to identity mismatch or other issues.

Throughout the process, any error or unsuccessful verification is immediately indicated by the red module and the voice prompt “recognition error” (Figure 3), allowing for timely correction. The PDA green module and voice prompt confirm the accuracy of identity verification. This process not only improves the efficiency of identity verification but also enhances caregivers’ alertness through color coding and voice prompts [19]. Additionally, new mothers and their families can actively participate in nursing safety management, increasing the sense of trust in the operation of nursing staff and ensuring the safety of both mother and child.

### Time Management of Newborns During Mother-Baby Separation

**Figure 4.**
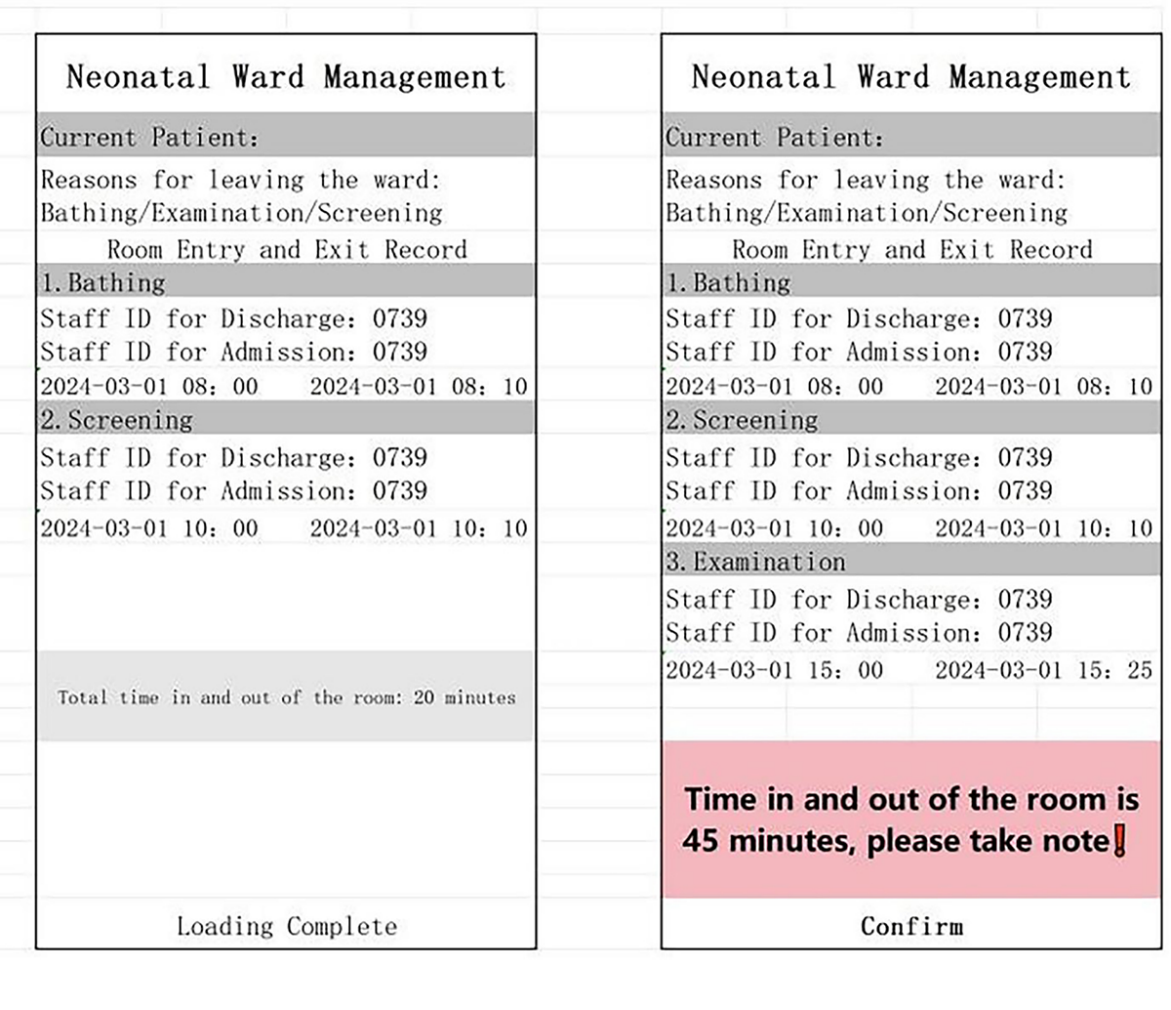
Key information on the management of neonatal admission and exit rooms.

The evaluation criteria of baby-friendly hospitals require that—except for the separation of mother and child with medical indications—the mother and newborn should remain together for 24 hours, with separation not exceeding 1 hour per day. Therefore, to support this requirement, time statistics and alarm functions relating to the newborn access room were added to the closed-loop management system (Figure 4). When the separation time exceeds the set threshold (ie, 45 min), the PDA immediately issues an alarm, to remind the nursing staff to send the newborn back to the room promptly.

Figure 4 provides key information on the management of neonatal admission and exit rooms, specifically including the following aspects:

1. Patient information display: the interface displays information about the current neonatal patient.
2. Reasons for leaving the ward: the system lists reasons for the newborn leaving the ward, including bathing, examination, or disease screening.
3. Time record: the exact time at which the newborn leaves and returns to the mother is recorded.
4. Staff identification: the identification number of the responsible staff member for the admission and exit of the newborn is recorded to facilitate tracking of the person.
5. Entry and exit time limit prompt: the interface particularly emphasizes that the time limit for newborns to enter and exit the room is 45 minutes and reminds the staff to pay attention to the time, so as not to exceed the specified time (ie, 1 h).
6. Warning tone function: if the mother-infant separation time exceeds 45 minutes, the system issues a warning tone, which helps the staff track the newborns and ensures the safety and standardization of the process.

### Statistical Analysis

The SPSS software (version 26.0; IBM Corp) was used for statistical processing. A paired design *t* test (two-tailed) was used for the comparison between the two groups. Categorical data were expressed as frequency (n) or percentage (%), and a *P* value of <.05 was considered statistically significant.

### Ethical Considerations

This study was conducted in accordance with the Declaration of Helsinki. This study was conducted with approval from the Ethics Committee of The Affiliated Suzhou Hospital of Nanjing Medical University (ID: KL901515). All family members of newborns gave their informed consent to this study. All data collected during this study were anonymized to protect the privacy and confidentiality of the participants. No personally identifiable information was retained in the dataset. The data were stored securely, with access restricted to the research team members who had undergone appropriate training in data handling and confidentiality. No financial compensation was provided to the participants for their involvement in this study. Participation was voluntary, and no incentives were offered. No images or supplementary materials containing identifiable features of the participants were included in the manuscript.

## Results

**Figure 5.**
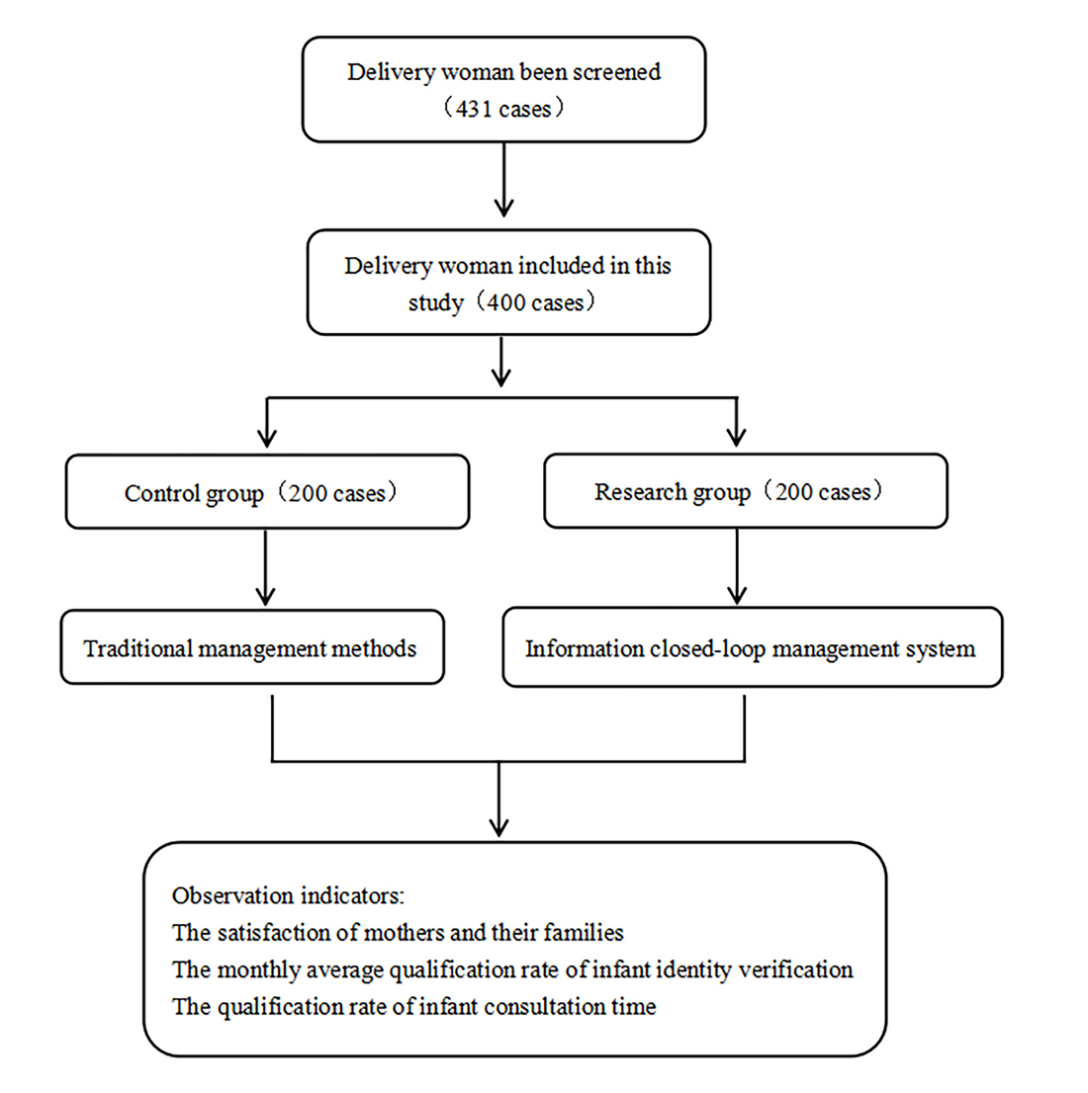
The participant screening process.

The participant screening process is shown in (Figure 5).

### Effect Evaluation

There were no significant differences in age, time of delivery, mode of delivery, economic status, educational level, or general characteristics between the study groups (*P*=.14).

### Identity Verification Pass Rate

Identity verification was performed at each entry, with the first verification result used as the assessment index for the identity verification pass rate. After implementation of the closed-loop process between July 2023 and May 2024, the average monthly pass rate of neonatal identity verification was 99.45 (SD 1.34), which was higher than that of the control group, 83.58 (SD 1.92) before the operation process (*P*=.02) (Table 1).

**Table 1.** Comparison of identity verification pass rate and satisfaction level between groups.

Variables	Control group (n=200), mean (SD)	Research group (n=200), mean (SD)	*P* value
Average monthly pass rate of neonatal identity verification	83.58 (1.92)	99.45 (1.34)	.02
Satisfaction level	92.82 (4.73)	96.45 (3.32)	.01

### Satisfaction Level

After the closed-loop process, the satisfaction among the pregnant women and their families was 96.45 (SD 3.32), which is higher than that of the control group, 92.82 (SD 4.73) before the closed-loop process (*P*=.01) (Table 1).

### Efficiency of the Entry and Exit Process

The efficiency of the traditional neonatal entry and exit process is low; for 80 newborns, a nurse must verify 240 labels daily, requiring approximately 80 minutes for copying and checking the labels. Compared to the traditional process, the closed-loop management system significantly reduced the execution time, decreasing the total verification time for 80 identity verifications to 15 minutes, with daily quality control verification requiring only 2 minutes. This improvement enhances the efficiency of neonatal nursing work and significantly improves the accuracy and efficiency of the verification work; thus, the process not only reduces the workload of nurses but also improves the quality of nursing services and ensures the safety of newborns. Before the implementation of the system, the exit and entry process could not effectively monitor the separation time between newborns and their mothers. After the implementation, the statistics of the background system showed that the real-time tracking and management of the newborn access times were achieved, with all newborns separations remaining below the 1-hour limit. These findings show that the newborn entry and exit times were effectively controlled to ensure their safety and timely nursing.

## Discussion

### Principal Findings

In this study, the monthly average qualification rate of infant identity verification after the implementation of the closed-loop process was higher than that of the control group. After the closed-loop process was implemented, the satisfaction of mothers and their families was higher than before the closed-loop process was implemented, and the separation time between infants and mothers was controlled within 1 hour.

### Innovation and Implementation Effect

The primary aim of closed-loop management is to ensure the safety of mother and child and improve the quality of care. In the existing literature, research on information closed-loop management systems for neonatal access rooms is relatively rare, particularly regarding changes in the implementation rate of identity verification standards before and after the implementation of the system, as well as statistical analyses of time monitoring within the access room. The closed-loop information management system developed in this study demonstrates significant innovation. Through the close collaboration between the interdisciplinary team, this system not only simplifies the traditional manual verification process but also ensures the accuracy and real-time performance of information.

Research has shown that intelligent systems can help practitioners make decisions, reduce their workload, and increase their efficiency and accuracy by incorporating artificial intelligence systems into their daily practice [20,21]. Regarding implementation effectiveness, a comparative analysis of the data before and after implementation, we found that the implementation rate of the newborn identity verification standards was significantly improved. The average monthly pass rate of neonatal identity verification in the study group was higher than that in the control group before the operational process, with a statistically significant difference. With precise time management, we ensured that newborns received timely and continuous care and improved the efficiency and response speed of care work. After the implementation of the system, 24-hour neonatal access room time supervision was achieved, and the qualification rate reached 100%, demonstrating high efficiency and systematic improvement in time management. Detailed statistical analyses of different ventricular event durations provided valuable data support. These data provided the basis for both the evaluation and improvement of current care processes and a scientific temporal database for future process optimization and the development of time management strategies. By analyzing these data, potential bottlenecks can be identified, the allocation of resources can be optimized, and the overall quality and efficiency of nursing services can be improved.

Closed-loop management methods have been explored in blood transfusion, breastfeeding, and care of premature newborns, yielding promising results [22-25]. Compared to traditional neonatal access management approaches, the closed-loop management system can not only reduce human interference factors but also trace the source of the whole process, strengthen supervision, further reduce risks, enhance doctor-patient relationships, and enhance trust, facilitate postpartum recovery, and reduce the risk of postpartum anxiety [26-28]. This closed-loop management system can also be extended to continuous blood glucose monitoring in critically ill newborns and pharmaceutical management for newborns, further enhancing full-process management and advancing precision medical digitalization [29,30].

### Improvements in Maternal and Infant Safety and Nursing Quality

The implementation of closed-loop management not only improves the safety of mothers and neonates but also significantly improves the quality of care. Through the data analyzed in this study, we found that after the implementation of closed-loop information management, the integrity of newborn access room records was significantly improved, and the traceability of nursing quality was enhanced. The completeness and traceability of data increased the safety of the mother and baby. Additionally, the intelligent system reduced the time burden on the nursing staff and provided convenient management of mother and baby information, which improved the quality of nursing and patient satisfaction. After the implementation of the system, caregivers’ time spent on identity verification decreased by 84.55%, which enabled them to devote more time and energy to direct nursing work, thus improving the overall quality of nursing services. Specifically, in the patient satisfaction survey, maternal and family satisfaction was significantly higher after the implementation of the closed-loop process than before, with a statistically significant difference.

### System Optimization and Prospects

Despite the system’s positive outcomes, there are still areas for improvement. For example, real-time monitoring, security alerts, and authentication need to be enhanced. In the future, we will continue to optimize information use to further refine nursing processes and improve the humanization and intelligence level of the system, to achieve more accurate and efficient nursing services. Additionally, we will also focus on how to achieve better closed-loop management and security measures through upgrading technology.

### Conclusion

The construction and application of an information-based closed-loop management system have enabled both real-time monitoring of newborns and comprehensive management of the entire nursing process. This system has significantly improved the safety of newborn care.

## Abbreviations

DBMS: database management system
PDA: personal digital assistant
